# Candida Prosthetic Valve Endocarditis

**DOI:** 10.7759/cureus.90752

**Published:** 2025-08-22

**Authors:** Sonia I Vicenty-Rivera, Alex P Rodriguez

**Affiliations:** 1 Research and Development, Veteran Affairs Caribbean Healthcare System, San Juan, PRI; 2 Cardiology, Bruce W. Carter Veterans Affairs (VA) Miami Healthcare System, Miami, USA

**Keywords:** aortic valve abscess, aortic valve disease, candida infection, fungemia, prosthetic valve endocarditis

## Abstract

Prosthetic valve endocarditis (PVE) represents a significant clinical challenge in cardiology and cardiac surgery. Characterized by infection of a prosthetic heart valve, PVE can lead to severe morbidity and mortality, necessitating a comprehensive understanding of its pathophysiology, epidemiology, clinical presentation, diagnostic methods, treatment strategies, and preventive measures. This case exemplifies the critical aspects of PVE, emphasizing its impact on patient outcomes and the broader implications for healthcare systems.

## Introduction

Prosthetic valve endocarditis (PVE) constitutes a significant clinical entity, characterized by considerable morbidity and mortality, and accounts for approximately 20% of all infective endocarditis cases. PVE arises from infection of prosthetic or surgically repaired cardiac valves, necessitating comprehensive evaluation of its epidemiology, clinical presentation, diagnostic modalities, therapeutic interventions, and preventive measures. The complexity of PVE underscores the imperative for heightened clinical vigilance and the development of optimized management strategies [1]. Non-cardiac complications frequently result from embolic phenomena, metastatic abscess formation, or the development of mycotic aneurysms. This case report critically appraises the salient features of PVE, including its pathophysiological mechanisms, wide-ranging clinical manifestations, and diagnostic complexities, while emphasizing its profound implications for patient outcomes and healthcare systems.

## Case presentation

This case involves a 45-year-old male patient, a non-smoker, with no illicit drug use, allergic to penicillin, with a prior history of a complicated hospital stay four months before. The patient had a prolonged hospital admission secondary to COVID-19 pneumonitis complicated with septic shock due to pneumonia with respiratory failure requiring endotracheal intubation, aortic valve bacterial endocarditis with severe valve regurgitation, and bacteremia with *Enterococcus faecalis*. There were other complications during his hospital stay, such as septic abscesses in the kidney and spleen, non-oliguric acute kidney injury requiring CRRT (continuous renal replacement therapy), and atrial fibrillation with a fast ventricular response.

The patient underwent aortic valve replacement with a bioprosthetic valve (22 mm pericardial tissue valve), a MAZE procedure, and left atrial appendage (LAA) ligation. A splenic drain was also placed to drain the abscess. The patient received treatment with multiple antibiotics, including vancomycin, meropenem, and daptomycin, administered intravenously. Renal parameters normalized to baseline (Cr 2.3 to 1 mg/dL). He was discharged home after six weeks of antibiotic treatment to continue physical therapy.

The patient continued physical therapy and had no further complications until four months after surgery, when he developed right upper quadrant pain and subjective fevers. The patient was also complaining of nausea and vomiting for three days and watery diarrhea. History was notable for sick contact with norovirus. The physical examination was remarkable for generalized tenderness to palpation, which was more pronounced in the right upper and lower quadrants, without guarding or rebound. No changes on cardiac auscultation with a soft systolic ejection 2/6 murmur with a clearly audible S2 at the aortic focus without a diastolic component. The examination of the extremities showed no skin or nail lesions suggestive of Jane's lesions or Osler's node lesions. The baseline laboratories showed a complete blood count with no leukocytosis, left shifting, or electrolytic abnormalities.

During admission, an abdominal CTS showed a bowel wall thickening in the cecum with pericecal inflammatory changes, lymphadenopathy, and terminal ileitis because of quantifiable febrile events of 100°F and above, blood culture, ova and parasites, HIV, CMV (*Cytomegalovirus*), *Clostridium difficile*, HTLV (human T-lymphotropic virus), Giardia AG, and *Cryptococcus*. All the laboratory parameters were negative, except for the blood culture analysis, which showed *Candida albicans* in multiple bottles. IV fluconazole (800 mg loading dose to continue 400 mg daily) was administered.

**Figure 1:**
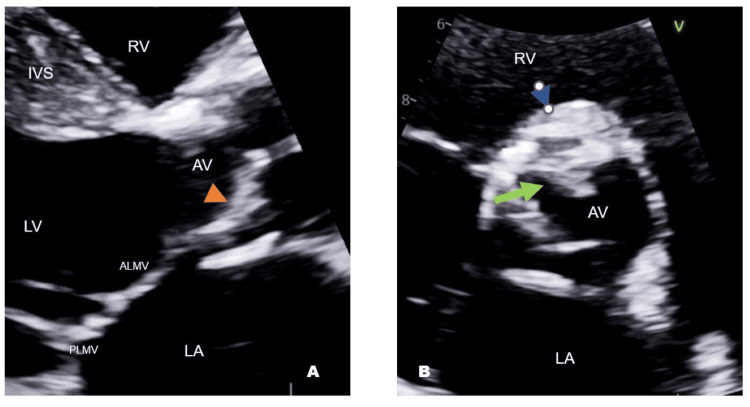
2D transthoracic echocardiogram, multiplane parasternal views of the bioprosthetic aortic valve. There is a thickening prosthesis with irregularly shaped, echo-dense masses with independent oscillatory motion more evidently attached to the right coronary cusp (arrows). ALMV: anterolateral mitral valve leaflet; PLMV: posterolateral mitral valve leaflet; LA: left atrium; RV: right ventricle; IVS: interventricular septum; LV: left ventricle

**Figure 2:**
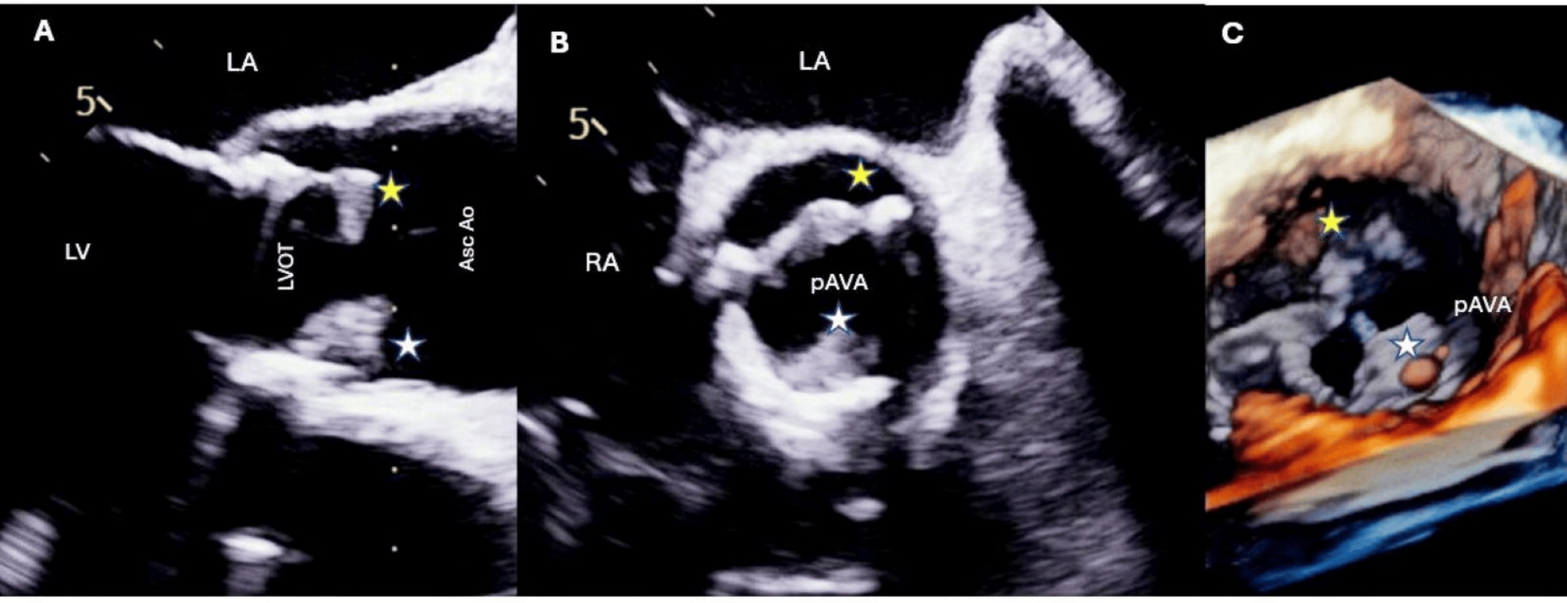
Transesophageal echocardiography revealed two mobile, irregularly shaped, echo-dense masses with independent oscillatory motion involving the anterior/right (yellow stars) and posterior/left (white stars) leaflets of the bioprosthetic aortic valve. The left panel (A) reflects the LVOT view at 120°, and the mid panel (B) reveals the short axis of the aortic valve view at 45–50%. The right panel (C) is a 3D image of the aortic valve demonstrating the bioprosthetic vegetations. LA: left atrium; LV: left ventricle; LVOT: left ventricle outflow tract; Asc. Ao: ascending aorta; pAV: prosthetic aortic valve

A 2D echocardiogram ruled out infected prosthetic valves because of fungemia. The study revealed an echogenic structure on the aortic valve annulus in close proximity to the RCC (right coronary cusp) base, as well as a small, mass-like structure on the ventricular side of the aortic bioprosthetic valve (Figure 1). To better characterize these lesions, a transesophageal echocardiogram was performed, which was notable for two vegetations on the ventricular side of the aortic valve, one on the RCC measuring 1.3 x 1.1 cm and the other on the noncoronary cusp (NCC) (4.5 mm x 7.0 mm) (Figure 2, Video 1). There was also an echo-lucent area on the annulus area at the base of the NCC, which could represent an early abscess or phlegmon formation at the AV (atrioventricular) annulus. After clinical evidence of Candida endocarditis, antifungal treatment was adjusted to micafungin 150 mg IV.

**Video 1:** Multiplane TEE 2D and 3D upper at 30 cm interrogation demonstrating two large vegetations—two mobile vegetations involving the anterior/right and posterior/left leaflets of the bioprosthetic aortic valve. TEE: transesophageal echocardiogram

**Figure 3:**
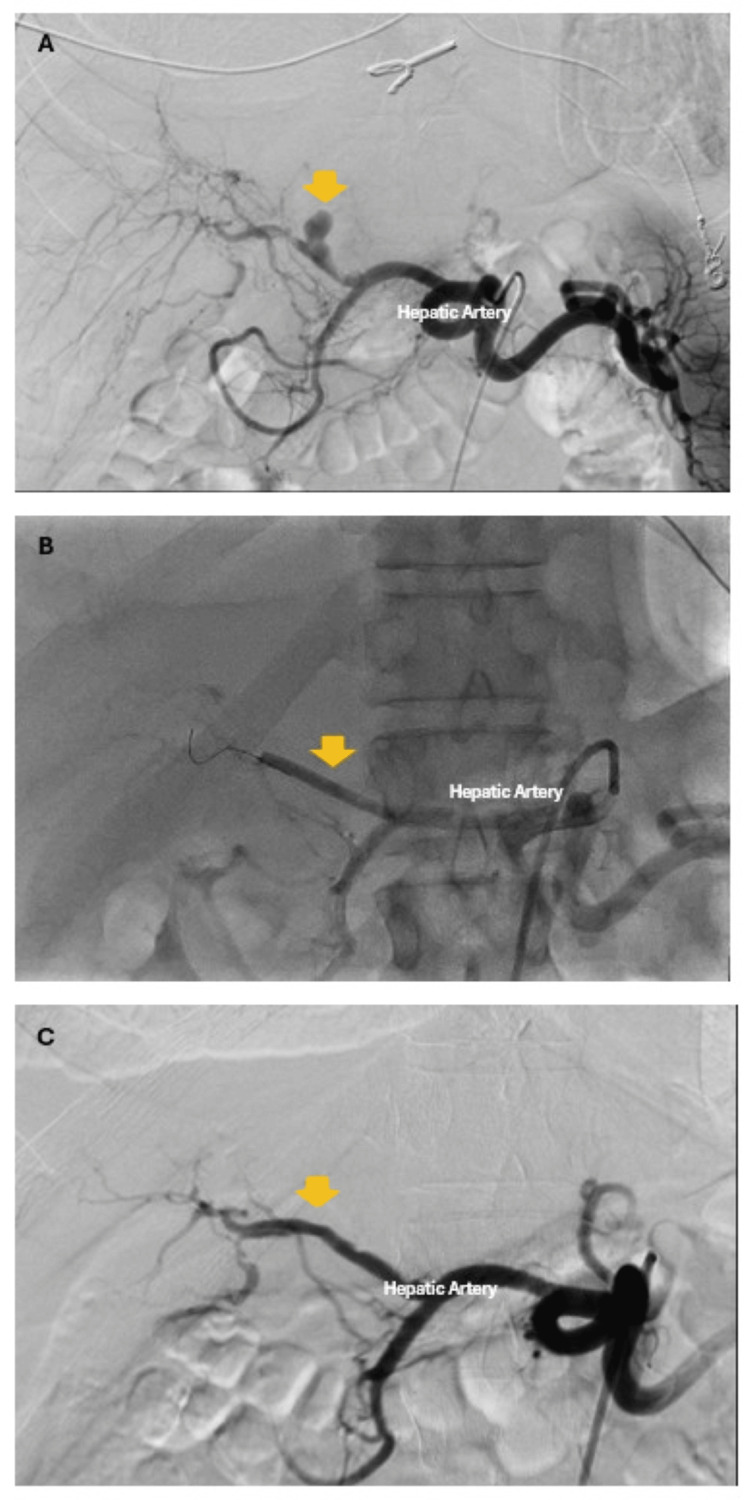
Angiography with balloon occlusion and thrombosis. (a) Angiography of the hepatic artery with an 11 x 9 mm saccular/fusiform aneurysm (yellow arrow); (b) 4 mm × 4 cm balloon (yellow arrow) placed overlying the hepatic aneurysm; (C) occlusion/thrombosis of the aneurysm (yellow arrow).

A CT angiogram was performed to rule out the possibility of septic embolism as an etiology for chronic abdominal pain. The study revealed persistent focal bowel wall thickening at the cecum, accompanied by stranding and adjacent adenopathy, as well as an area of nodular soft tissue density encasing/attenuating a few branches of the mid superior mesenteric artery, and abutting the superior mesenteric vein, which is concerning for a possible mycotic aneurysm. Also, there was an area at the intrahepatic bile ducts with periportal edema and amorphous soft tissue concerning a 1.1 cm mycotic pseudoaneurysm arising from the left or right hepatic artery, with a surrounding abscess and small peripheral wedge-shaped areas in the spleen and a focal area of hypodense heterogeneity with mild stranding at the medial left kidney, which may reflect infarcts (Figure 3). Interventional radiology performed a CTA-guided thrombosis with balloon occlusion of the hepatic artery pseudoaneurysm.

Even though the patient had Candida endocarditis, the cardiothoracic service recommended postponing aortic valve replacement for a later time because of friable aortic valve tissue after recent surgery and present endocarditis, which would be considered high risk for valve dehiscence and complications during the postoperative period. Therefore, they recommended that the patient continue with intravenous antifungals until completion of complete intravenous treatment before surgical valve replacement. By then, the patient was to be transitioned to oral antifungal therapy with isavuconazonium indefinitely as suppressive therapy. Fluconazole was not considered a possible choice because it prolongs the QT segment, and the patient already had a 12-lead ECG with a prolonged QT interval (500 ms) secondary to chronic antifungal treatment.

The patient was treated with intravenous antifungal medication with blood clearance for fungi. After six weeks of antifungal intravenous therapy, the patient underwent redo bioprosthetic aortic valve replacement (26 mm aortic valve homograft; LifeNet Health, Virginia Beach, VA) with mitral valve repair (30 mm FlexForm Band; Genesee BioMedical, Denver, CO) and surgery for superior mesenteric artery mycotic aneurysm repair. The patient remains on isavuconazole for long-term suppression after initial treatment due to increased concern of possible recurrence of infection.

## Discussion

PVE occurs when an implanted cardiac valve or other non-CIED (cardiac implantable electronic device) intracardiac foreign bodies develop an infection. It has been reported in up to 20-30% of tertiary medical centers, with 36% of cases involving transcatheter aortic valve (TAVI) and 20% of surgically implanted valves [2,3]. PVE is classified into two main presentations, depending on the timing of infection after valve surgery [4,5]. When PVE occurs within the early postoperative period (less than 60 days after surgery), it is primarily associated with organisms that are part of the skin flora, such as Staphylococcus aureus, coagulase-negative *Staphylococcus* species (e.g., *S. epidermidis* and *S. haemolyticus*), gram-negative bacilli, and *Candida *species*.* On the other hand, late PVE develops more than 60 days after the procedure and often involves organisms more commonly associated with community-acquired native valve endocarditis, including *Streptococcus viridans* and* Enterococcus *species. However, *Staphylococcus aureus* and coagulase-negative Staph species can also contribute to late PVE cases.

Building on the classification of PVE, it is essential to recognize that patients may present with clinical manifestations similar to native valve endocarditis (fever, chills, and heart murmur) and a similar diagnostic process that will include blood cultures (microbiologic data), cardiac imaging studies, and clinical criteria. However, diagnosing PVE requires identifying the infecting organism through culture, serologic, or molecular testing and identifying valvular vegetations, paravalvular abscesses, or prosthetic valve structural complications on cardiac imaging studies. While bacterial endocarditis is more frequently recognized, fungal endocarditis, mainly caused by *Candida* species, has gained increasing attention due to its rising incidence and associated morbidity and mortality [3,4]. The incidence of PVE varies with valve type, patient population, and risk factors, with the highest risk in the first year post-surgery. PVE occurs in approximately 1-6% of patients with prosthetic valves. Candida infective endocarditis (CIE) comprises only 1-2% of all infective endocarditis (IE) cases but carries a high mortality rate (30-80%). Increasing cardiac surgeries and risk factors underscore the need for awareness, especially in patients with prolonged hospital stays, intravenous drug use, or compromised immunity [5,6]. Embolic complications may be one of the first and only indicators of CIE.

Candida PVE pathophysiology involves the interaction between host factors and the virulence traits of *Candida* species [7]. Patients with prosthetic heart valves are at an increased risk of developing endocarditis due to disruptions in the endothelial surface, which allow for microbial colonization. *Candida* species, especially *Candida albicans*, have several virulence factors, including the ability to form biofilms on prosthetic devices. These biofilms protect the organisms from host immune responses and antifungal treatments. The presence of biofilms complicates the clinical management of PVE, making it resistant to surgical intervention and systemic antifungal therapy [7].

Given the underlying mechanisms, recognizing the clinical presentation of PVE, whether insidious or acute, is crucial for timely intervention. In the case of Candida PVE, symptoms often emerge gradually and can be nonspecific. Common symptoms include fever, chills, fatigue, and heart murmurs [6-8]. Non-cardiac complications are usually a result of an embolic event, metastatic abscess formation, or a mycotic aneurysm. Distinctive features may consist of embolic phenomena, which can lead to systemic complications such as stroke, renal failure, or splenic abscesses. Additionally, patients might show signs of heart failure or exhibit new heart murmurs, indicating valvular dysfunction. The diagnosis of Candida PVE may be delayed due to the nonspecific nature of these symptoms and the difficulties associated with identifying fungal pathogens in blood cultures. Therefore, a high index of suspicion and early diagnosis are crucial for effective management [8].

Effective recognition of symptoms leads to the next step: accurate and timely diagnosis of PVE, which is crucial for effective management of the condition. The modified Duke criteria serve as a widely accepted framework for diagnosing endocarditis, incorporating clinical, microbiological, and echocardiographic findings. Blood cultures remain the gold standard for identifying the causative organism, but transthoracic and transesophageal echocardiography play a vital role in visualizing vegetation and assessing valve function. Echocardiography, especially transesophageal echocardiography, is crucial in diagnosing PVE, as it can visualize the vegetations on the valve, a key diagnostic feature. In some cases, advanced imaging techniques like cardiac magnetic resonance (CMR) imaging or cardiac computed tomography (CCT) may be employed to provide additional information. Managing PVE, especially Candida PVE, is complex and requires prompt diagnosis. Blood cultures may be negative in up to 50% of fungal cases, delaying diagnosis and treatment. Serologic tests and imaging, including echocardiography, are crucial in identifying infections, underscoring the need for enhanced diagnostic strategies [7-9].

The management of Candida PVE is complex and usually requires a multimodal approach. The choice of therapy is guided by culture results and susceptibility testing, with prolonged courses often necessary due to the biofilm-forming ability of many organisms involved in PVE. Antifungal therapy typically involves the use of echinocandins (e.g., caspofungin or micafungin) or triazoles (e.g., voriconazole) [8,9]. The choice of antifungal agent depends on the specific *Candida* species and its susceptibility profile. Surgical intervention is often necessary, particularly in cases of severe valvular dysfunction, abscess formation, or persistent infection despite antifungal therapy. Surgical options may include valve replacement and debridement of infected tissue. The timing of surgery is crucial, as it can significantly impact patient outcomes. Underscoring the importance of multidisciplinary collaboration among cardiologists, cardiac surgeons, and infectious disease specialists in formulating an effective treatment plan, highlighting the value of teamwork in patient care [9,10].

Preventive strategies are paramount in reducing PVE incidence. Prophylactic antibiotics are recommended for high-risk patients, and education on oral hygiene and chronic disease management further helps mitigate risk [8,9]. Additionally, antifungal prophylaxis benefits select populations, and stringent infection control measures are essential in healthcare settings. Providers should maintain a strong suspicion for fungal endocarditis, especially in those with prosthetic heart valves.

## Conclusions

PVE constitutes a formidable clinical challenge in contemporary cardiovascular medicine, necessitating a nuanced and multidisciplinary approach that encompasses microbiological, clinical, and surgical considerations. The increasing incidence of Candida PVE, attributable to the growing prevalence of prosthetic heart valves and cardiac devices, further complicates the diagnostic and therapeutic landscape. Early detection of atypical clinical presentations and vigilance for embolic phenomena are paramount, as these may serve as critical harbingers of Candida endocarditis. This case underscores the imperative of heightened clinical suspicion and timely intervention to mitigate adverse outcomes.

Timely identification and management of PVE are essential to improving clinical outcomes and minimizing both morbidity and mortality. Optimal patient care is predicated upon the concerted efforts of a multidisciplinary team, including infectious disease specialists, cardiologists, and cardiac surgeons, to devise and implement individualized therapeutic strategies. Such collaborative approaches are crucial for achieving favorable prognoses in this high-risk patient population.
